# pH Colorimetric Sensor Arrays: Role of the Color Space Adopted for the Calculation of the Prediction Error

**DOI:** 10.3390/s20216036

**Published:** 2020-10-23

**Authors:** Andrea Pastore, Denis Badocco, Sara Bogialli, Luca Cappellin, Paolo Pastore

**Affiliations:** Department of Chemical Sciences, University of Padua, Via Marzolo 1, 35131 Padua, Italy; andrea.pastore@unipd.it (A.P.); denis.badocco@unipd.it (D.B.); sara.bogialli@unipd.it (S.B.); luca.cappellin@unipd.it (L.C.)

**Keywords:** colorimetric sensors, pH measurement, Hue, cationic surfactant, *OrMoSil*

## Abstract

A pH colorimetric sensor array was prepared and characterized by combining tetrabromophenol blue (TBB) and bromothymol blue (BB) embedded in organically modified silicate (*OrMoSil*) spots polyvinylidene fluoride (PVDF)-supported. The signal was based on the *Hue* profile (*H*). The individual calibrations of TBB and BB showed precisions with minimum values of 0.012 pH units at pH = 2.196 for TBB and 0.018 at pH = 6.692 for BB. The overall precision of 10 spots of the mixture TBB/BB increased in the pH range of 1.000–8.000 from a minimum value of pH precision of 0.009 at pH = 2.196 to 0.012 at pH = 6.692, with the worst value of 0.279 pH units at pH = 4.101. The possibility to produce an array with much more than 10 spots allows for improving precision. The *H* analytical performance was compared to those of other color spaces such as RGB, Lab, and XYZ. *H* was the best one, with prediction error in the range of 0.016 to 0.021 pH units, at least three times lower than the second-best (x coordinate), with 0.064 pH units. These results were also confirmed by the calculation of the main experimental contributions to the pH prediction error, demonstrating the consistency of the proposed calculation approach.

## 1. Introduction

Colorimetric sensor arrays (CSAs) [1] are chemical sensors using suitable dyes to detect a specific analyte [2,3,4,5,6,7,8,9]. Color variations are usually recorded with CCD cameras or scanners [10]. The red, green, blue (RGB) color space is widely used in colorimetric sensing processes, but the composition of the R, G, and B does not change monotonically with spectral wavelength and intensity [11]. In 1931, the Commission International de l’Éclairage (CIE) defined the concept of the tristimulus values X, Y, and Z based on the three-component theory of color vision. The receptors of the human eye are responsible for three primary colors (red, green, and blue), and all colors are mixtures of them. The XYZ tristimulus values are obtained by using suitable color matching functions. The Lab model, indirectly obtained from the CIE-XYZ color space, is made of two chromatic components (a and b) and a lightness component (L). The two models express a wider gamut than the RGB. Ideally, they can reproduce an infinite number of chromatic mixtures [12]. Other color spaces are characterized by a specific tone or hue, a saturation level, and a lightness component [12]. The H component of the HSV (hue, saturation, value) is more stable and robust than the other color spaces as the illumination is enclosed in the V component of the model [11,13,14,15]. Nevertheless, the HSV model has some issues. The first occurs when the maximum and minimum values for RGB are the same, which corresponds to the gray tones (undefined value for hue). This causes some incorrect color interpretations. The second issue occurs when the maximum and minimum RGB values sum two (saturation value undefined) [16]. The analytical performance of a CSA is strongly affected by the choice of chemo-responsive dyes [17,18]. It is possible to tailor the Hue transition of a pH indicator with a second dye, but the identification of the effective enhancement condition is not trivial [19,20]. Another important aspect is the composition of the matrix in which the indicator molecule is entrapped. Recently, the *OrMoSil* (organically modified silicate) hybrid matrix was developed [21,22]. The presence of Si-C non-hydrolyzable bonds partially avoids leaching phenomena [23], although it is not sufficient [24]. An important improvement comes from the use of cationic surfactants [19,24,25]. Indeed, there is an important effect of the counterion and the alkyl chain length of the cationic surfactant on the reversibility and working interval of the pH sensor [26]. Most parts of the pH sensors described in the literature are characterized by precision in the range 0.10–0.56 pH units and the response time, in some cases, up to 15 min, not comparable to the analytical performance of a pH-meter [13,14,27,28,29]. Recently, we demonstrated that the precision error can be lowered by at least one order of magnitude, rendering this kind of device suitable for robust quantitative analysis [30].

In the present paper, an *OrMoSil* PVDF-supported colorimetric sensor will be used for monitoring the error behavior. The analytical performance of the H coordinate was compared to those of other color spaces such as RGB, Lab, and XYZ by quantifying the pH prediction error. Indeed, the three main error contributions, ε, β, and δ, affecting a generic color coordinate, i.e., the pH prediction error calculated with the same coordinate, will be numerically estimated. β is the background level due to the lighting conditions and to the support, δ is the error due to the image acquisition conditions and ε is the instrumental error of the camera. The validation of the proposed error theory will be done through the comparison of the results belonging to two independent series of spots containing tetrabromophenol blue (TBB) or bromothymol blue (BB) to achieve robust results. Finally, we will estimate the overall precision of a pH CSA based on a suitable combination of two pH indicators, TBB and BB, with a working range of pH 1.000–8.000.

## 2. Materials and Methods

### 2.1. Reagents and Instrumentation

Dodecyltriethoxysilane, TEOS (Tetraethyl Orthosilicate) (≥99%), HCl 37%, tetrabromophenol blue (TBB, 85%), bromothymol blue (BB, 95%), hexadecyltrimethylammonium *p*-toluenesulfonate (CTApTs), acetic acid, and NaOH (≥97%) were purchased from Sigma Aldrich, whilst KCl was purchased from Prolabo. Sodium hydrogen carbonate (99.8%), sodium dihydrogen phosphate, and absolute ethanol were provided by Carlo Erba. We illustrated the cell used for pH measurements in our recent paper [13]. A Crison MM 40 pH-meter and a combined glass electrode (calibration with two standard solutions Mettler Toledo; pH = 6.865 and 4.006) were used for the reference pH measurements. Analytical (AS 220 R2 Radwa) and technical (EU-C500 Gibertini) balances were used for weight measurements. The pH buffers have a 0.1 M total concentration. The color of the wet spots was sampled in the most homogeneous portion of the spot (≈120 pixels). Background detection occurs in an external area near the spot. Dedicated programs written with MATLAB were employed to figure out the color coordinates. The regressions were obtained by using the iterative algorithm “Levenberg Marquardt” [31].

### 2.2. Preparation of the CSA

**Figure 1:**
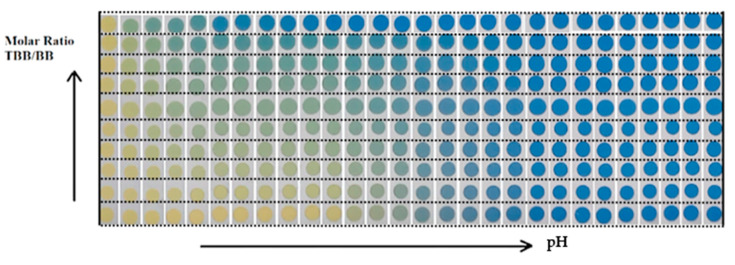
Pictures of the 10-spot CSA sensor, deposited by hands, with various molar ratios of tetrabromophenol blue/bromothymol blue (TBB/BB). Molar ratio and pH increase in the direction of the arrows. The colors come from the immersion of the sensor in 28 pH buffers from pH 1 to pH 10.

The preparation of the *OrMoSil* sol was made by mixing 4.03 g of TEOS, 0.65 g of dodecyl-TEOS, 1.58 g of Milli-Q water, and 0.55 g of 0.03 M HCl. To prepare the 10-spot sensor, CTApTs was now added (1.75 g) together with TBB/BB in the following molar ratios: 0.024, 0.061, 0.098, 0.147, 0.184, 0.233, 0.331 and 0.478, respectively. The tenth spot (first row in Figure 1) contained only TBB. The spots were aged at 20 ± 2 °C for three days before use. After a prior conditioning cycle, the pH CSA was immersed consecutively, for 100 s, in each buffer solution (28 pH values) from the acidic pH interval to the basic one.

## 3. Theoretical Considerations

### 3.1. Main Error Contributions Affecting a Generic X Color Coordinate

The pH value measured with a CSA requires a suitable camera able to read the color. The color space usually adopted is the sRGB. Nevertheless, this color space is not the best in terms of stability, robustness, and precision of the signal [11]. In the following sections, the analytical performance of the *H* coordinate from *H*SV color space will be compared to those of other color spaces such as RGB, Lab, and XYZ. The best performance of *H* has been already cited by other authors, although no-one, to our knowledge, has rationalized its behavior [11,13,14]. The quantitative rationalization will be based on the pH prediction errors. Since the variance of the color coordinate affected the overall prediction error, the choice of the color space plays an important role. For this reason, it will be determined a ranking of the best performing coordinates. If *X* is a generic experimental color coordinate and μ is its theoretical value, we can write:

X = μ(X) + β + δ + ε(X).(1)

The parameters β, δ, and ε are error sources defined as follow:

- β is the background level due to the lighting conditions and to the CSA support (associated with the spot);
- δ is the error due to the image acquisition conditions (associated with the spot);
- ε is the instrumental error of the camera (associated with the detected color).

In particular, it will be demonstrated that *H* is affected only by the ε contribution.

### 3.2. Linearization of the Sigmoidal Calibration Model

The nature of an acid-base indicator is to change its color at the p*K*_a_ value. The color transition is usually sigmoidal and can be managed with *X*. The calibration function that interprets the *X* vs. pH profile of a single pH indicator is given by the usual Boltzmann equation:

(2) $$X\left(\mathrm{pH}\right)=X_{In}+\frac{\left(X_{HIn}-X_{In}\right)}{1+e^{4\left(\mathrm{pH}-{\mathrm{pH}}_{i}\right)/\Delta \mathrm{pH}}}$$

where *X_HIn_* and *X_In_* are the *X* color values of the *HIn* and *In* forms, respectively. ΔpH is the pH working interval of the indicator (the interval in which is possible to observe a variation of the color coordinate). This parameter is a function of the indicator but also (as we will see below) of the chosen color coordinate. The pH_i_ parameter is the pH value of the inflection point. Δ*X*$=\left|X_{In}-X_{HIn}\right|$ is the *X* maximum variation. The sensitivity is obtained by considering the ratio: $SL_{X}=\Delta X/\Delta \mathrm{pH}$. Mixtures of two indicators required a bi-sigmoidal model:

(3) $$X\left(\mathrm{pH}\right)=X_{0}+\Delta X\left[\frac{p}{1+e^{4\left(\mathrm{pH}-{\mathrm{pH}}_{i,1}\right)/\Delta {\mathrm{pH}}_{1}}}+\frac{1-p}{1+e^{4\left(\mathrm{pH}-{\mathrm{pH}}_{i,2}\right)/\Delta {\mathrm{pH}}_{2}}}\right]$$

where *X*_0_ is the initial *X* value. The parameters *p* and 1 − *p* represent the contribution of the two indicators to the *X* value; pH_i,1,_ and pH_i,2_ are the pH values of the first and the second inflection point; ΔpH_1_ and ΔpH_2_ are the working intervals around the first and the second inflection point of the bi-sigmoid. Since the variance of *X* for some indicators is not homoscedastic in the transition zone [19,30], the sigmoidal regression must be weighted. For this reason, it was convenient to linearize the Boltzmann sigmoidal equation to obtain a homoscedastic calibration interval simplifying the calculation of the discriminated pH accuracy [30]. The linearization is the following:

$$F=ln\frac{X_{HIn}-X}{X-X_{In}}=a+b\cdot \mathrm{pH}$$

where *a* and *b* are the intercept and slope so that the working interval of the indicator is ΔpH=4/b and the inflection point is ${\mathrm{pH}}_{i}=-a/b$. The error of the discriminated pH is given by:

(4) $$s_{\mathrm{pH}}=s_{y/x}\;{\left[1+\frac{1}{n}+\frac{{\left(\mathrm{pH}-\bar{\mathrm{pH}}\right)}^{2}}{\sum {\left({\mathrm{pH}}_{i}-\bar{\mathrm{pH}}\right)}^{2}}\right]}^{\frac{1}{2}}$$

where s_y/x_ is the regression standard deviation.

## 4. Results and Discussion

Before starting the discussion, we wish to point out that since the glass electrode was used to calibrate our CSA, our devices cannot give better results than the potentiometric technique. The pH errors calculated for our sensors are, in some cases, of the order of few thousandths of pH units. These values are extreme even for potentiometric measurements; therefore, the reported results will demonstrate only the comparability of our CSA with the glass electrode, although the CSA can have better precision.

### 4.1. Experimental Analytical Performance of Various Color Spaces

**Figure 2:**
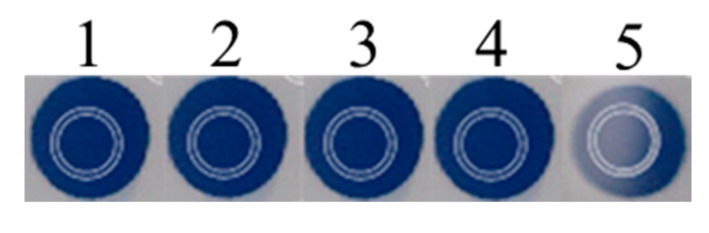
Five repeated BB spots. The fifth spot shows a light reflection area caused by a non-optimal cell geometry. The white circles indicated the color sampling area (120 pixels).

In this section, the experimental analytical performance of the *H* coordinate was compared to those of other color spaces such as RGB, Lab, and XYZ. We focused our attention on five repeated BB spots which are nominally identical. One of them showed a light reflection area caused by a non-optimal cell geometry (see Figure 2, spot 5).

This situation was chosen on purpose to evaluate the influence of anomalous signals on the overall result. Table 1 summarizes the results achieved from the sigmoidal profiles of the color coordinates (*X*) vs. pH in terms of $s_{{\mathrm{pH}}_{i}}$ calculated with Equation (4) at the inflection point.

Data were ordered with increasing prediction error ($s_{{\mathrm{pH}}_{i}}$). The performance of *H* was the best one in all spots. In particular, $s_{{\mathrm{pH}}_{i}}$ was in the range 0.016–0.021 pH units, at least three times lower than the second-best (the x coordinate), with a pH error in the interval of 0.064–0.109 pH units. The *H* coordinate exhibited the smallest regression variance and the lowest ΔpH (0.9 pH units). None of the other coordinates gave comparable results. Concerning the fifth spot affected by reflection phenomena (see Figure 2), it can be fitted only by using the *H* coordinate. In other cases, the spot was unusable. On the other hand, the other coordinates are less sensitive but work in a wider pH range, sometimes larger than two logarithmic units. By using the *H* coordinate, the spot response was less affected by the spot shape, concentration, and optical inhomogeneity, and it maintained the same value ($s_{s_{{\mathrm{pH}}_{i,H}}}$ = 0.002;$\;s_{s_{{\mathrm{pH}}_{i,B}}}$ = 0.005;$\;s_{s_{{\mathrm{pH}}_{i,G}}}$ = 0.011; $s_{s_{{\mathrm{pH}}_{i,R}}}$ = 0.012;$\;s_{s_{{\mathrm{pH}}_{i,L}}}$ = 0.013; $s_{s_{{\mathrm{pH}}_{i,y}}}$ = 0.013; $s_{s_{{\mathrm{pH}}_{i,x}}}$ = 0.019). Moreover, leaching or re-arrangements of the pH indicator in the spot did not alter the *H* value. A significant consequence is that only the calibration obtained with *H* remains identical in time.

### 4.2. Quantification of the Error Contributions on pH Discrimination

In this section, the precision of the pH value obtained with *H* will be evaluated and compared to the other color coordinates. *H* is defined as [19]:

(5) $$H=\left[\frac{D}{\Delta }+n\right]\frac{1}{6}$$

**Figure 3:**
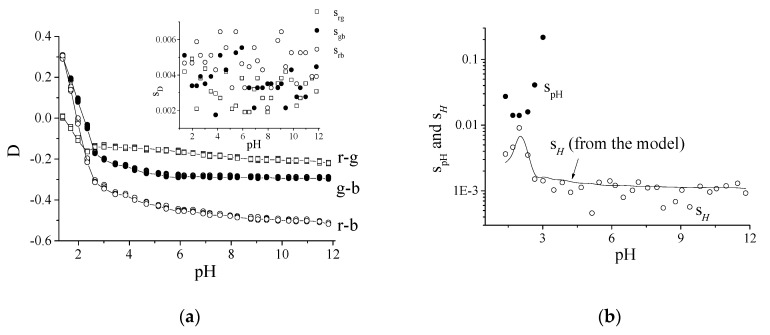
(**a**) Experimental r-g (□), g-b (●), and r-b (○) vs. pH profiles of 5 independent spots of TBB in OrMoSil. Insert: standard deviations with pH. (**b**) Experimental s*_H_* (○) and s_pH_ (●) variation of the same spots vs. pH. The continuous line represents the s*_H_* theoretical value obtained from the cited model.

It contains D, which is the function difference of the normalized coordinates, r − g, g − b, or r − b (r = R/255, g = G/255, b = B/255), and Δ is the product between luminance and saturation [19,30]. As *H* contains a difference function, on the basis of Equation (1), we can write:

*H* = μ(*H*) + ε’(*H*).

where ε’ represents the error associated with a couple of rgb coordinates considered. It is evident that the sum β + δ elides for D as its value is identical for the rgb coordinate of the same spot but it will not elide for the other *X* coordinates. To calculate the error contributions, β, δ, and ε, the overall variance of D (i) and a generic *X* coordinate (ii) were calculated. In particular, (i) the experimental $s_{D}^{2}=2s_{\varepsilon }^{2}$ is obtained considering the average variance of the experimental values r-g, g-b, and r-b where β+δ does cancel; (ii) $s_{X}^{2}=2s_{\beta \delta }^{2}+2s_{\varepsilon }^{2}$ is obtained when all the contributions to the variances of the rgb coordinates were considered so that β+δ does not cancel. Figure 3a reports D vs. pH (r-g (□), g-b (●), and r-b (○)) of five independent spots of TBB. The insert of Figure 3a reports the average standard deviations (i) of the same spots,$\;s_{D}$. They were constant with pH and similar: $s_{r-g}=0.0031$, $s_{g-b}=0.0035$ and $s_{r-b}=0.0047$ (*p*_value_ < 0.001) so that $s_{D}≅s_{\Delta }$. On the other hand, from (ii), the variance of *X*, with the couple r-g, taken as an example, was: $s_{X}^{2}=s_{r}^{2}+s_{g}^{2}\;$. These errors are larger—0.0090, 0.0060 and 0.0085—for $s_{r,g}$, $s_{g,b}$ and $s_{r,b}$, respectively, as expected.

The estimation of the errors for the five repeated spots of TBB was:

$$s_{\varepsilon }^{2}=\frac{s_{D}^{2}}{2}=\left(\frac{s_{r-g}^{2}+s_{g-b}^{2}+s_{r-b}^{2}}{3}\right)/2=7.3\cdot 10^{-6}\;\mathrm{for}\;(i)$$

and

$$s_{\beta \;+\;\delta \;+\;\varepsilon }^{2}=\frac{s_{X}^{2}}{2}=31.5\cdot 10^{-6}\;\;\mathrm{for}\;(\mathrm{ii})$$

respectively. The ratio between these two values is very close to the one calculated from the data in Table 1 between *H* and x coordinate (0.23 ≈ 0.24), indicating the correctness of the error calculation. Figure 3b reports the experimental standard deviations, s*_H_* (○) and s_pH_ (●) vs. pH, referring to the same spots. The continuous line, in good agreement with the experiment, is the theoretical s*_H_* profile (Equation (5) in [19]).

### 4.3. Calculation of the Discriminated pH Precision for a CSA

To estimate the overall precision of a CSA, $s_{{\mathrm{pH}}_{w}}$, the weight of each spot was $w_{j}=\frac{1}{\sigma_{{\mathrm{pH}}_{i}}^{2}}$. By using the same estimate of $s_{H_{p}}$ for all the spots, the weighted ${\mathrm{pH}}_{w}$ was:

$${\mathrm{pH}}_{w}=\frac{1}{T}\overset{nspot}{\underset{i=1}{\sum }}\frac{{\mathrm{pH}}_{i}}{\sigma_{{\mathrm{pH}}_{i}}^{2}}=\frac{\sum_{i=1}^{n}{\left(\Delta_{j}S_{H_{j}}\right)}^{2}{\mathrm{pH}}_{i}}{\sum_{i=1}^{n}{\left(\Delta_{j}S_{H_{j}}\right)}^{2}}=\overset{n}{\underset{i=1}{\sum }}w_{j}\cdot {\mathrm{pH}}_{i}$$

where Δ_*j*_ is the product of the saturation by the luminance of the *j*th spot and $S_{H_{j}}$ is the sensitivity  ΔH/ΔpH of the *H* sigmoidal profile of the *j*th. The sum of the weights *T* is:

$$T=\sum_{i=1}^{nspot}\frac{1}{\sigma_{{\mathrm{pH}}_{i}}^{2}}=\frac{3}{4s_{Hp}^{2}}\sum_{i=1}^{n}{\left(\Delta_{j}S_{H_{j}}\right)}^{2}\;\mathrm{and}\;w_{j}=\frac{{\left(\Delta_{j}S_{H_{j}}\right)}^{2}}{\sum_{i=1}^{n}{\left(\Delta_{j}S_{H_{j}}\right)}^{2}}$$

The corresponding error is:

$$s_{{\mathrm{pH}}_{w}}=\frac{1}{T}=\frac{1}{\sqrt{\sum_{i=1}^{n}\frac{1}{\sigma_{{\mathrm{pH}}_{i}}^{2}}}}=\frac{2}{\sqrt{3}}\frac{s_{H_{p}}}{\sqrt{\sum_{i=1}^{n}{\left(\Delta_{j}S_{H_{j}}\right)}^{2}}}$$

The parameter $w_{j}$ was set to 0 when the calibration sensitivity is less than 0.1 so that the pH measurement is centered within the most sensitive calibration zone.

### 4.4. The Behavior of the Mixture TBB/BB in the CSA

**Figure 4:**
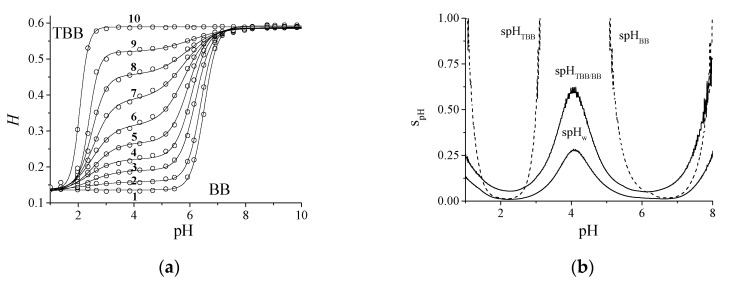
(**a**) Circles, experimental calibration curves relative to 10 polymeric spots of TBB (curve 10), BB (curve 1), and their mixtures with molar ratio *n_TBB_/n_BB_*: 0.024, 0.061, 0.098, 0.147, 0.184, 0.233, 0.331 and 0.478 (curve 2–9). Continuous line, bi-sigmoidal fitting between pH 1 and 10. (**b**) Experimental profiles of the precisions, $s_{\mathrm{pH}}$, obtained from calibration curves 1 ($s_{{\mathrm{pH}}_{\mathrm{BB}}})$, 5 ($s_{{\mathrm{pH}}_{\mathrm{TBB}/\mathrm{BB}}})$, 10 ($s_{{\mathrm{pH}}_{\mathrm{TBB}}})$ and the overall ones, $s_{{\mathrm{pH}}_{w}}$.

The circles in Figure 4a describe 10 *H* calibration profiles (28 pH values each, from pH 1 to 10) obtained with TBB and BB at different molar ratios and the continuous lines are the corresponding bi-sigmoidal curve fitting. The shift from TBB to BB is evidenced. Both the acidic and alkaline plateau of TBB alone (curve 10) and BB alone (curve 1) are coincident, as both have complementary color transitions (yellow-blue). The use of the TBB/BB mixture allows for widening the pH interval, as they have different p*K*_a_ values. Figure 4b reports the experimental profiles of the $s_{\mathrm{pH}}$ precisions referring to curves 1 ($s_{{\mathrm{pH}}_{BB}})$, 5 ($s_{{\mathrm{pH}}_{TBB/BB}})$, 10 ($s_{{\mathrm{pH}}_{TBB}})$ and the overall one ($s_{{\mathrm{pH}}_{w}})$. Prediction errors of 0.012 pH units at pH = 2.196 and 0.018 pH units at pH = 6.692 were obtained with the data of curves 10 (TBB) and 1 (BB), respectively. The spot relative to curve 5 (both indicators present) had the worst precision at pH 2.2 and 6.7 compared to the curve of the single indicators TBB (curve 1) and BB (curve 10) since the bi-sigmoidal plot decreases the slope at the inflection points. On the other hand, the zone of pH 2.600–6.100 was improved. The minimum TBB precision error obtained with the calibration of a single spot was comparable with 0.014, reported in Figure 3b, relative to five independent spots, indicating a repeatable deposition procedure.

By considering all the 240 pH data in Figure 4a in the pH range 1–8, an improvement of the overall precision is evident: $s_{pH_{w}}=\;$0.009 pH units at pH = 2.196 and $s_{pH_{w}}=\;$0.012 pH units at pH = 6.692. The most critical pH region, close to pH 4.101, was also significantly improved: $s_{pH_{w}}=\;$0.279 pH units at pH = 4.101. The production of arrays with more than 10 spots produces a further improvement of the precision. Table 2 reports the calibration parameters of curves 1–10. The *H*_0_ and the ∆*H* values were constant for all the mixtures.

## 5. Conclusions

A colorimetric sensor array (CSA) to detect pH based on tetrabromophenol blue (TBB) and bromothymol blue (BB) will be used for monitoring the relevant error behavior. The analytical performance of the H coordinate was compared to those of other color spaces, such as RGB, Lab, and XYZ by quantifying the pH prediction error. The pH prediction error, $s_{{\mathrm{pH}}_{i}}$, obtained with *H* was in the range 0.016 to 0.021 pH units, at least three times lower than the best of the other coordinates (the *X* coordinate from CIE-XYZ color space was characterized by a 0.064 pH units error). The ratio between the variances of the difference coordinate D and a generic *X* color coordinate is the same as that emerging from the pH prediction error calculated on *H* and the *X* coordinate, indicating the correctness of the error calculation. In particular, the use of *H* eliminated the error contributions coming from the spot preparation and background anomalies (β) and the lighting conditions (δ). The performance of *H* was, therefore, the best one, since it is affected only by the instrumental error due to the camera characteristic, ε. This kind of CSA is characterized by errors of the same order of the potentiometric technique in the indicator working interval. The overall precision $s_{{\mathrm{pH}}_{w}}$ of only 10 spots of TBB/BB mixture in the pH range 1.000–8.000 has minimum error values of $s_{{\mathrm{pH}}_{w}}=$0.009 at pH = 2.196 and of $s_{{\mathrm{pH}}_{w}}=\;$0.012 at pH = 6.692, with the worst precision at pH = 4.101, $s_{{\mathrm{pH}}_{w}}=\;$0.279. In any case, the possibility to produce arrays with much more than 10 spots allows for improving precision.

## Figures and Tables

**Table 1:** Values of ΔpH, R^2^ and$\;s_{{\mathrm{pH}}_{i}}$ referred to the linear fitting, F, of the best performing coordinates (first and sixth columns). Data referred to 5 independent BB spots, the fifth of which showed a light reflection area.

*X*	Spot #	ΔpH	R^2^	$s_{pH_{i}}$	*X*	Spot #	ΔpH	R^2^	$s_{pH_{i}}$
*H*	5	0.9	0.999	0.016	x	3	2.2	0.996	0.080
*H*	3	0.9	0.999	0.017	L	2	2.2	0.996	0.081
*H*	2	1.0	0.999	0.017	y	2	2.1	0.995	0.084
*H*	4	0.9	0.999	0.020	R	2	2.3	0.996	0.085
*H*	1	1.1	0.999	0.021	G	2	2.1	0.994	0.090
X	1	2.0	0.997	0.064	G	4	2.2	0.994	0.096
Y	1	2.0	0.996	0.070	B	4	2.6	0.995	0.097
L	1	2.1	0.996	0.070	R	3	2.4	0.995	0.097
R	1	2.2	0.996	0.073	L	4	2.4	0.994	0.100
G	1	2.0	0.996	0.074	y	4	2.3	0.994	0.101
G	3	2.1	0.996	0.075	B	1	2.6	0.995	0.101
L	3	2.2	0.996	0.076	B	2	2.6	0.995	0.104
X	2	2.1	0.996	0.076	B	3	2.5	0.994	0.109
Y	3	2.2	0.996	0.077	x	4	2.4	0.993	0.109

**Table 2:** Fitting parameters *H*_0_, ΔH, *p*, pH_1_, pH_2_, R^2^, ΔpH_1,_ ∆pH_2_ obtained with a bi-sigmoidal regression (Equation (3)) of experimental data of Figure 4a.

r	*H*_0_	ΔH	*p*	pH_1_	pH_2_	R^2^	ΔpH_1_	∆pH_2_
BB	0.136	0.456	0.000	2.58	6.52	0.9996	2.38	0.92
0.024	0.130	0.460	0.068	2.14	6.39	0.9999	2.18	0.97
0.061	0.130	0.456	0.135	2.44	6.21	0.9995	2.00	1.13
0.098	0.130	0.457	0.205	2.39	6.08	0.9991	1.87	1.20
0.147	0.132	0.453	0.300	2.55	5.96	0.9987	1.65	1.31
0.184	0.130	0.456	0.407	2.50	5.81	0.9993	1.59	1.70
0.233	0.135	0.452	0.556	2.56	5.80	0.9992	1.34	2.02
0.331	0.139	0.447	0.706	2.49	5.87	0.9990	1.04	2.13
0.478	0.138	0.450	0.845	2.38	6.06	0.9987	0.74	2.05
TBB	0.139	0.450	1.000	2.06	6.00	0.9994	0.62	1.85
